# Costly Genes

**DOI:** 10.1371/journal.pgen.1008889

**Published:** 2020-08-20

**Authors:** Maria Karayiorgou

**Affiliations:** Columbia University, Department of Psychiatry, New York, New York, United States of America; University of California Los Angeles, UNITED STATES

Each time I give a lecture about finding causes of mental illness, I am asked “How long before a treatment?” My answer is usually “5–10 years,” because that always seems like it would be enough time. Starting in the late ‘80s, molecular biology revolutionized gene identification for a number of diseases, especially monogenic ones [1–4]. A psychiatrist at that time, I felt inadequate treating mental illness with the existing drugs, none of which were informed by causality. So, I decided to train in molecular biology in order to figure out the genetics of mental illness with the ultimate goal to one day develop better treatments–treatments based on the underlying genetic susceptibility of each affected individual. The path to gene identification, and from there to treatment, is long, arduous, and uncertain. First, we have to show (on a molecular level) that genes are involved, then find out how they are involved, then identify individual genes and what their function is, and finally figure out how we, the scientists, can intervene. A tall order, but one worth spending a lifetime on, I thought.

A huge success story in the late ‘80s was the cloning (identification) of the cystic fibrosis gene, CFTR (cystic fibrosis transmembrane conductance regulator) [5]. Cystic fibrosis (CF) affects 100,000 patients worldwide (30,000 in the US alone), and is a monogenic disease, albeit with multiple mutations in that gene. The defective gene causes a buildup of thick mucus in the lungs, making it very difficult to breathe. CF is a life-threatening disease with no cure [6]. So, the gene identification was an important finding, and was celebrated as such. I remember it well. Fast forward to now, 30 years later, and we are finally ready to capitalize on the promise of finding the CFTR gene; a new treatment called Trikafta, which targets the most common CF mutations, was recently developed and approved by FDA. This drug is the first triple combination therapy and could help about 90 percent of CF patients [7–9]. Since it is specific to causation (targeting the disease-causing genetic mutations), it has the potential to be a cure rather than just a treatment for patients carrying these mutations. In the very least, it can transform CF from a deadly disease to a chronic condition. This is a very exciting development indeed, and although it took 30 years since the discovery of the gene (a clear departure from the socially-tolerable answer of 5–10 years), it is nevertheless a hugely welcomed development for the field and, most importantly, for those suffering from and affected by this terrible disease.

But there’s a catch: the drug will cost more than $300,000 a year. The reason for this is not price gauging or greed from pharma, but rather the small number of patients who will actually use the drug. What worries me is that this will not be a problem unique to a rare disease like CF, which affects only 100,000 people worldwide, but will alarmingly be the case for some of the most common diseases that each affect a staggering 1–10% or more of the population worldwide; mental illness, Alzheimer’s, Parkinson’s disease, heart disease, obesity, hypertension, and diabetes all fall in this category. Pharma determines pricing by taking into account how much money they spent to develop a drug divided by the number of people in need of this particular drug divided by the number of years that the pharma holds the patent while generics cannot interfere with their sales. The more we delve into the molecular genetics of common diseases, the more it looks like the second factor of the pharma equation (i.e. the number of people with genetically homogeneous druggable disease subtypes who would respond best to a given drug) will be small. As our chances to develop better, causality-based drugs increase, the number of target patients for each of those drugs decreases, creating a novel landscape in medicine. Up until now, when pharma introduced a new drug for a common disease (let’s use depression as an example), they expected it to be prescribed to virtually everyone suffering from depression—5–10% of the entire worldwide population. It did not matter that the drug was not effective for all patients. Instead, it was seen as the best available option, and it was prescribed widely. This scenario created a clear path to profit for pharma, and justified their hard, costly work in developing this drug in the first place. Now, however, with the advances of molecular biology, human genome sequencing, and ensuing successes in gene identification, we have arrived at a different place. As we discover more of the genetically homogeneous, rare subgroups of all these common disorders, we should not have to worry about our ability to take the logical and necessary next step: developing drugs that could cure each subgroup and making them affordable and available to people who need them. We all need to wake up and see the new reality of medicine: each common disease will look like a jigsaw puzzle of causes, due to multiple mutations, and treating each subgroup *will* be taxing.

This does not mean that we should give up our efforts to find causes, nor does it mean that lives of people with less-common diseases do not matter. It only means that we need to adapt. Let’s use the CF story as an example to plan ahead, think through the challenges that the new landscape of molecular medicine presents, get organized, and make the necessary changes. In order for personalized, precision medicine to live up to its promise and allow the development of causality-based treatments which have the potential to be cures for their intended users, we need to be creative and incentivize pharma. We need pharma to not be discouraged, but stay in the fight and do the hard work that is needed to follow and see-through the hard work of the scientists discovering the genes. Some examples of such incentivization could be to extend the patent duration for small-market drugs so that pharma can have enough time to make back the money they spent; or reward pharma that develops a successful small-market drug with tax breaks; or make insurances cover a larger percentage of the cost for small-market drugs. If we, as a society, cannot address the new norm of medicine, the daunting jigsaw puzzle of treatments, then it was all for nothing. My own work and the work of hundreds of other scientists who devoted our lives to finding genes for the diseases that ail so many of us was all for nothing. Currently, a significant percentage of tax payers’ money (the NIH budget) is being used to fund gene discovery research. If this research does not and cannot translate into affordable, useable treatments that can save lives, then what is the point?

If the current pandemic has taught us anything, it is that nature is a beast that must be respected, and if humans want to stay ahead of its curve, we need to invest in science. And I’m not talking about the “last minute hallelujah” investment for a “miracle drug,” I’m talking about investing and planning methodically, steadily, and with resolve, knowing that the incredibly difficult and painstaking work required to take a problem apart will not, at the end of the day, get dropped by shortsightedness, indifference, lack of leadership, vision, or creativity, or profit-mongering and other human faults. The ultimate goal is too important for that, and it demands and requires attention by governmental and other regulatory agencies. Every life matters. These new, causality-based drugs that will be developed, although they will be small-scale drugs, have a good, solid chance of being cures. We should all want that. Anything less is criminal.
